# Determination of Physicochemical and Functional Properties of Coconut Oil by Incorporating Bioactive Compounds in Selected Spices

**DOI:** 10.1155/2020/8853940

**Published:** 2020-07-28

**Authors:** Dilini N. Perera, Geeth G. Hewavitharana, S. B. Navaratne

**Affiliations:** Department of Food Science and Technology, University of Sri Jayewardenepura, Nugegoda 10250, Sri Lanka

## Abstract

Lipid oxidation has been identified as a major deterioration process of vegetable oils, which leads to the production of primary and secondary oxidative compounds that are harmful to human health. Oleoresins of ginger, garlic, nutmeg, pepper, cloves, and cinnamon were extracted and incorporated into coconut oil, and change occurrence on physicochemical properties, thermal stability, shelf life, and antioxidant activity was monitored against the same properties of pure coconut oil. Lipid oxidation was assessed in terms of the free fatty acid level and peroxide value. For the comparison purpose, another oil sample was prepared by incorporating vitamin E too. Results revealed that both peroxide value and FFA of pure and flavored coconut oil samples after a one-week storage period were 3.989 ± 0.006 and 3.626 ± 0.002 mEq/kg and 0.646 ± 0.001 and 0.604 ± 0.002 (%), respectively. Saponification value, iodine value, smoke point, and the flashpoint of flavored oil were decreased while increasing the viscosity during storage. The highest phenolic content and DPPH free radical scavenging activity were found in flavored coconut oil. Since spices containing antioxidants, the thermal stability of flavored oil was better than that of pure coconut oil. Both oleoresins and vitamin E-incorporated samples showed the same pattern of increment of FFA and peroxide value during storage; however, those increments were slower than those of pure coconut oil.

## 1. Introduction

Fats and oils play a major role in the human diet. Apart from that, it also affects the sensory qualities of processed foods. Coconut oil is one of the widely used cooking oil in many Asian countries [1]. Over the last decade, the world production of coconut oil has been increased because of its important edible characteristics. In the context of edibility, there are two types of coconut oil, RBD (Refined, Bleached, and Deodorized) and the virgin. However, both contain similar fatty acids and a triglycerol profile [2]. Lima and Block [2] reported that coconut oil contains 92% saturated fatty acids (SFA); of that, 62% of FAs have the carbon chain length between 8 and 12. The major fatty acid of coconut oil is lauric which is a medium-chain fatty acid (MCFA) [3]. MCFA are important as they act as inert sources of energy and are easier to absorb, metabolize, and store in the body [4]. As coconut oil is composed of more saturated FAs, it is more resistant to oxidation and polymerization than the oils with unsaturated fatty acids [2].

Lipid oxidation is a major deterioration process in cooking oils. It affects organoleptic properties as well as the keeping quality of oil-related food products. Several methods including genetic modifications, compositional changes via chemical means, and addition of synthetic antioxidants like TBHQ, BHT, and BHA have been practiced in order to improve the stability of edible oils [5]. Synthetic antioxidants are reported to be health hazardous, and some are removed from the GRAS (generally recognized as safe) list and banned in many countries. Therefore, natural antioxidants such as oleoresins and volatile oils from spices and herbs have attracted a lot of attention in lipid oxidation. The addition of different spices and herbs to cooking oils was a traditional practice to enhance the aroma and taste of food particularly in Mediterranean cuisines [6]. Thus, they incorporated different spices and herbs such as ginger, garlic, nutmeg, cloves, cinnamon, and pepper for their recipes. Therefore, they were able to make products with better shelf life and consumer preference due to the presence of natural antioxidants as well as an attractive flavor profile [6]. In keeping of those in mind, this study was conducted to develop flavored coconut oil by incorporating oleoresins of spices and analyzing effectiveness of it in terms of physicochemical properties, thermal stability, shelf life, and antioxidant activity against the same of pure coconut oil (without incorporation of oleoresins).

## 2. Materials and Methods

A commercial coconut oil supplied by the Marina Oil Company in Sri Lanka (SL) was used for the experiments. Oleoresins of ginger, garlic, nutmeg, cloves, pepper, and cinnamon were purchased from a reputed internationally registered supplier in SL.

### 2.1. Preparation of Flavored Oil

An oleoresin mixture was prepared by adding ginger : garlic : cinnamon : nutmeg : cloves : black pepper into a 1 : 1 : 1 : 1 : 1 : 1 ratio (0.001 g). The mixture was blended with oil thoroughly, and it was kept in dark brown-colored bottles and stored under ambient condition (27°C) for the subsequent use of the study.

### 2.2. Determination of Physicochemical Properties of Oils

Chemical properties such as peroxide value (AOCS Cd 8b–90) [7], FFA (AOCS Cd 3d-63) [7], iodine value (AOCS Cd 1b-87) [7], and saponification value (AOCS Cd 3-25) [7] and physical properties such as moisture (AOAC 925.10) [8], insoluble impurity percentage (IUPAC 2.604) [9], smoke point (AOCS Cc 9a-48) [7], flashpoint (AOCS Cc 9a-48) [7], specific gravity (AOAC 920.212) [8], color (Chroma Meters CR-410C), and viscosity (AOAC 22.00) [8] were measured according to the standard protocols aforementioned.

### 2.3. Determination of Thermal Stability of Flavored Coconut Oil

The stability of oil was analyzed at 170°C for 2 hours by taking 10 g of oil samples for every 30 minutes, and collected samples were subjected to determine peroxide value (PV) and FFA level.

### 2.4. Storage Stability of Flavored Coconut Oil

Storage stability of oils was assessed by resorting the rapid aging test. Therein, flavored oil as well as pure coconut oil samples were taken, and each oil was filled into 36 glass bottles and stored in a hot air oven at 60°C for twelve weeks. A positive control of coconut oil in 36 bottles with vitamin E (without oleoresins) was also kept under the same condition for comparison purposes. Samples were withdrawn at seven days of intervals for twelve weeks, and storage stability was analyzed in terms of peroxide value and FFA level.

### 2.5. Total Phenolic Content of Oil

The total phenolic content of oil was analyzed according to the method of Redondo-Cuevas et al. [10] with slight modifications. Therein, 100 *μ*l aliquot of each sample was transferred to a test tube, and it was mixed with 5.80 ml of distilled water, 500 *μ*l Folin-Ciocalteu reagent, and 1500 *μ*l of sodium carbonate. The mixture thereafter was vortexed for 30 s and incubated at 40°C for 30 minutes. The absorbance of the mixture was measured at 760 nm wavelength using a Uv-Vis spectrophotometer (Camspec, Model M550, True Double Beam). Gallic acid was used as the standard to generate a calibration curve. Total phenolic content was expressed as a gallic acid equivalent using a linear equation based on the calibration curve.

### 2.6. Determination of DPPH Radical Scavenging Activity (Antioxidant Assay)

To analyze the antioxidant activity of coconut oil, the method described by Pradhananga and Manandhar [11] was used with slight modifications. Thus, 500 *μ*l aliquot of each concentration was mixed with 2500 *μ*l of DPPH working solution in screw-capped 4 ml micro test tubes covered with an Al foil. Thereafter, the mixture was vortexed for 30 seconds and left to react for 30 minutes in the dark. Finally, the absorbance of oil was measured at 517 nm wavelength using a spectrophotometer.

### 2.7. GC-MS Analysis for Flavored Coconut Oil

Sodium methyl ester of oil samples was prepared and transferred into the GC valves. For the GC analysis, a HP-5ms nonpolar column was used and the temperature was programmed from 80°C to 200°C at the rate of increment 5°C/min during analysis. Helium was used as the carrier gas, and internal pressure was maintained at 100 kPa. The injector temperature was 250°C.

### 2.8. SPME-GC-MS Analysis for Volatile Compounds

Two grams (2 g) of the flavored coconut oil sample was placed into a 20 ml GC vial, tightly capped with polytetrafluorethylene (PTFE) septum, and left for 3 hours at 30°C to allow the equilibration of the volatiles in the headspace. After equilibration, the septum covering each vial was pierced with an SPME needle, and the fiber was exposed to the headspace for 40 minutes. The volatiles adsorbed fiber was thermally desorbed in the hot injection port of a gas chromatograph. Helium was used as the carrier gas, at a flow rate of 0.9 ml/min, and the temperature was programmed from 80°C to 200°C at the rate of increment 3°C/min during analysis.

### 2.9. Statistical Analysis

Statistical analyses were performed with Minitab statistical package (version 17). The mean separation was performed using Fisher's Least Significance Difference (LSD) with a 95% confidence level. All the measurements are the mean value of triplicates.

## 3. Results and Discussion

### 3.1. Physicochemical Properties of Oil

Pure and flavored coconut oil samples were analyzed concerning the following physicochemical properties, and results are given in Table 1.

According to the data given in Table 1, PV and FFA of pure coconut oil after one week of storage were 3.989 ± 0.006 mEq/kg and 0.646% ± 0.001, respectively, and the values for the same parameters of flavored coconut oil in the same order were 3.626 ± 0.002 mEq/kg and 0.604% ± 0.002. The statistical analysis revealed that the *p* value of both samples for FFA and PV was less than 0.05 (*p* < 0.05). Therefore, there was a significant difference between FFA and PV of flavored and pure coconut oil samples. The FFA level and PV of flavored coconut oil were decreased compared with pure coconut oil during storage. This may be due to the presence of oleoresins of spices which may have acted as natural antioxidants, and thus, they may prevent the oxidation of fatty acids [12]. According to Ghosh et al. [13], the FFA level and PV of coconut oil extracted from West coast tall variety, cultivated in Kerala, India, were 0.08 ± 0.02 and 2.89 ± 0.02, respectively, and these values were somewhat incompatible with the values obtained from this study.

Iodine numbers are often used to determine the amount of unsaturated fatty acids in oils. Unsaturated fatty acids are usually recommended for healthy consumption over a high percentage of saturated fatty acids in oils. However, highly unsaturated fatty acids in oils undertake an oxidative degradation process due to double-bond configuration unless enough antioxidant is added [14]. According to the data given in Table 1, the iodine value of both pure and flavored coconut oils was 6.452 ± 0.08 and 5.081 ± 0.02, respectively. Since the *p* value of both samples was less than 0.05 (*p* < 0.05), there is a significant difference between the iodine value of flavored and pure coconut oil samples. This is maybe due to the diminishing of the double bonds in polyunsaturated fatty acids in coconut oil by chemical energy of oleoresins [15].

The saponification value (SV) of oil directly correlates with the average molecular weight of all the fatty acids present in it. Most of the mass of fat/triesters have 3 fatty acids with different chain lengths. Long-chain fatty acids found in the fats have a low saponification value because they have a relatively fewer number of carboxylic functional groups per unit mass of the fat as compared to short-chain fatty acids [16]. The results of this study (Table 1) show that the mean SVs of pure and flavored coconut oils were 249 ± 1 and 222 ± 1 mg KOH/g, respectively, and the *p* value of all samples was less than 0.05 (*p* < 0.05). Therefore, there is a significant difference between the saponification value of flavored and pure coconut oil samples. According to Codex Alimentarius [17] and the APCC Standards [18], the SV value of the coconut oil is in the range of 250-260 and 248-268 mg KOH/g oil, respectively. The SV is related to the mean molecular mass of the fats and oils, and it is inversely related to the chain length of the fatty acids. This means that the higher the SV, the shorter the average chain length of fatty acids [19]. However, in this study, one gram of flavored coconut oil contained a considerable number of oleoresins. Therefore, SV of flavored coconut oil gets a lower value, and this value is significantly lower than (*p* < 0.05) that of pure coconut oil.

The low moisture content is a requirement for a long storage life of oils; because moisture in oil causes hydrolyzation of triglycerides initially thereafter, it causes rancidity [14]. The moisture contents of all flavored and pure coconut oil samples of this study were within the range of 0.1-0.2%. As per the Quality Standards of Technical Information & Typical Analysis of the Australian Oilseeds Federation [20], the maximum allowable moisture content in edible oils shall be ≤0.2%. And it is for the edible coconut oil as per the Sri Lanka Standard Institution [21] 0.1%. According to this study, the moisture content of flavored oil samples was increased after one week of storage. However, this increment was not significant because the calculated *p* value of both samples was greater than 0.05 (*p* > 0.05). The reason for this phenomenon was based on the ethanol base oleoresin extraction as it did not contain free water at all [22].

In insoluble impurities of flavored coconut oil which is the same as pure coconut oil samples (0.05 ± 0.01%), the *p* value of all samples was higher than 0.05 (*p* > 0.05) after one week of storage. Therefore, there is no significant difference between insoluble impurities in flavored and pure coconut oil samples.

The smoke point of cooking oil is an important factor because heating of oil to a particular point may cause the oil to liberate smoke which may contain toxic fumes and harmful free radicals. It is defined as the loss of small molecular fragments due to evaporation [23]. The smoke point and flashpoint of pure coconut oil subjected to this study were 193°C ± 3 and 222°C ± 2.6, respectively, while these values for the flavored coconut oil in the same order were 157°C ± 2.5 and 181°C ± 1. According to the results, the smoke point and the flashpoint of all the flavored coconut oil samples were decreased, and the *p* value of all samples was less than 0.05 (*p* < 0.05). Therefore, there is a significant difference between the smoke point and the flashpoint of flavored and pure coconut oil samples. Since boiling points of oleoresins are lower than the smoke point of coconut oil, the smoke point of oleoresin-incorporated flavored oil turned lower [24].

The viscosity of oils is depending on the nature of the triglycerides present in it. The viscosity changes due to the different arrangements of the fatty acids on the glycerol backbone of the triglyceride molecule. Therefore, viscosity is related to the chemical properties of the oils such as chain length and saturation/unsaturation [25]. The viscosity of pure and flavored coconut oil samples of this study after one week of storage was 18.633 ± 0.05 and 19.4.00 ± 0.05 mPa·s, respectively. According to the results given in Table 1, the viscosity of flavored oil was increased after the incorporation of oleoresins. Since the *p* value of all samples was less than 0.05 (*p* < 0.05), there is a significant difference between the viscosity of flavored and pure oil samples. The reason for the increment of the viscosity of the flavored sample was the addition of oleoresins, which are usually somewhat viscous [26].

The specific gravity of pure and flavored coconut oil samples was 0.924 ± 0.005 and 0.995 ± 0.005, respectively. According to the results, the specific gravity of flavored oil was increased in comparison to the pure coconut oil, and the *p* value of all oil samples was less than 0.05 (*p* < 0.05). Therefore, there is a significant difference in the specific gravity of flavored and pure coconut oil samples. The reason for the increment of specific gravity of flavored oil samples was the addition of oleoresins, which are usually compact and dense comparatively pure oils [24]. According to Ghosh et al. [13], the specific gravity of coconut oil extracted from West coast tall variety, cultivated in Kerala, India, was recorded as 0.92 ± 0.01. Hence, the finding of Ghosh et al. [13] is tallied with the finding of this study.

After incorporation of oleoresins, the color of coconut oil was increased from pale yellow to dark brown while decreasing *L* value from 72 ± 2 to 67 ± 2. Hence, the darkness of flavored oil has been increased considerably. Further, redness (*a*∗) of flavored oil has been increased from 5 ± 1 to 14 ± 1 while decreasing yellowness (*b*∗) from 69 ± 2 to 46 ± 2. Therefore, when increasing the redness of flavored oil, the yellowness reciprocally comes down. Since the *p* value of all samples was less than 0.05 (*p* < 0.05), there is a significant difference between the color of flavored and pure coconut oil samples. The reason for the color change of flavored coconut oil was adding the oleoresin mixture which was usually dark in color. Apart from that, heating also caused to produce many decomposed products that are also dark in color. Similar findings have been reported in a color change of oils subjected to heating due to the accumulation of nonvolatile decomposition products such as oxidized triacylglycerol and FFA that can lead to color changes [5] which also indicates the quality deterioration of oil too.

### 3.2. Thermal Stability of Flavored Oil Compared to the Pure Oil

Since oils are considered as the medium for frying of many food products, the oils must be thermally stable at the frying temperatures. Thermal degradation of oils at frying temperature results in several chemical reactions which include hydrolysis, oxidation, thermal decomposition, and polymerization [27]. Nevertheless, prolonged heating also reduces the organoleptic and nutritive quality of oils. Under this circumstance, the thermal stability of the flavored oils at 170°C was evaluated in terms of FFA and PV by this study.

FFAs are formed by the breakdown of triacylglycerol on hydrolytic or autoxidation. It has been reported that on thermal processing, hydrolysis occurs more in oil with short-chain fatty acids than oil with long-chain saturated fatty acids [28]. According to some research studies, when temperature maintains at 170°C of the flavored oils, they exhibited better stability with a very small increase in FFA as compared to the original oil samples [5].

**Figure 1:**
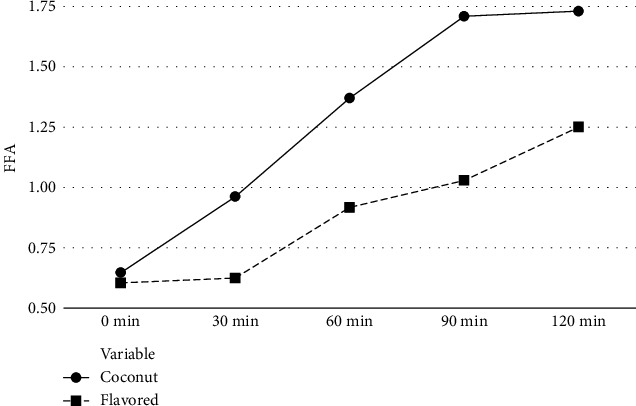
FFA level changes with frying time.

According to Figure 1, FFA was found to be increasing with the increment of heating time of all oil samples subjected to this study. Pure coconut oil samples exhibited the highest FFA (even if the temperature was maintained at 170°C for 0, 30, 60, 90, and 120 min); when the temperature was maintained at 170°C for 120 minutes, it was 1.730 ± 0.01. However, for the flavored coconut oil, it was 1.250 ± 0.01 under the same conditions. Further, when the high temperature of hot oil is maintained for a long time, an increment of FFA has also occurred correspondently; however, the rate of increment of flavored oil was lower. In this study, since the calculated *p* value of all samples was less than 0.05 (*p* < 0.05), there is a significant difference between FFA of flavored and pure coconut oil samples (at all temperatures).

**Figure 2:**
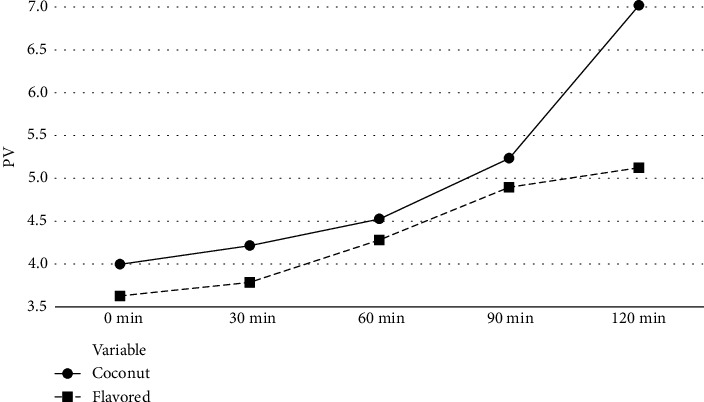
PV changes with frying time.

PV of oil samples was also increased with the increment of heating time, and a high PV was observed in pure coconut oil samples compared with flavored oil samples (Figure 2). Conversely, the rate of increment of PV of flavored oil samples was lower than that of pure coconut oil samples. Further, the calculated *p* value of all oil samples was less than 0.05 (*p* < 0.05); there was a significant difference between the peroxide value of flavored and pure coconut oil samples.

The lesser PV of flavored oil may be due to the presence of antioxidants in the essential oils of pepper, garlic, cinnamon, nutmeg, and ginger that can quench the initiation and propagation steps of autooxidation reactions [29]. Karoui [30] reported that there was a sharp rise in the peroxide value when the temperature was raised from 25 to 180°C during the heating of corn oil. They also reported that corn oil with thyme extract showed the least peroxide value during deep frying. A similar study was conducted by Pradhananga and Manandhar [11]. They have also reported that FFA and PV of sunflower oil were found to be increasing with the heating time and reported that FFA and PV can be decreased by adding TBHQ. Similar results were found by Singh et al. [31] and Hou et al. [32], with their studies with different types of oils.

### 3.3. Shelf Stability of Flavored Coconut Oil against Pure Oil

The oxidative stability of coconut oil samples was measured in terms of PV and FFA for 12 weeks by storing them at 60°C. The oxidative deterioration of oils is significantly faster when stored in the elevated temperature while exposing it to light rather than storing under in-house conditions [33].

**Figure 3:**
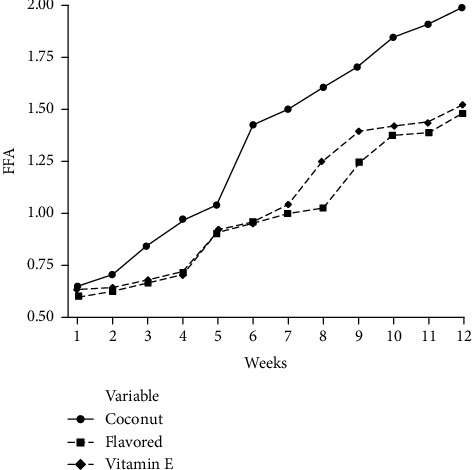
Changes of FFA level in coconut oil samples during storage.

The presence of FFA in oil is an indication of insufficient processing, lipase activity, or other hydrolytic actions. According to Figure 3, it is evident that the initial FFA level of the pure coconut oil sample was slightly higher than that of the flavored sample. In the accelerated storage at 60°C for 12 weeks, a slow and steady increment of FFA has occurred in all the samples. Pure coconut oil samples exhibited the highest FFA level during the whole 12-week period of storage, and it was highest (1.998 ± 0.01) in the 12th week (Figure 3). In the case of flavored and vitamin E-added coconut oils, FFA levels at the 12th week of storage were 1.491 ± 0.01 and 1.530 ± 0.01, respectively. The FFA level of all oil samples was increased with the storage time, but the rate of increment in flavored and vitamin-E added oil was rather lower than that of the pure coconut oil samples (Figure 3). Since the *p* value of pure and flavored oil samples was less than 0.05 (*p* < 0.05), there was a significant difference between FFA levels of flavored and pure coconut oil samples. However, there was no significant difference between FFA levels of flavored and vitamin E-added samples because the *p* value was higher than 0.05 (*p* > 0.05). Changes in the FFA level of this study were similar to the result obtained by Iqbal and Bhanger [34]. They had used sunflower oil and garlic extract in stabilizing the oil at accelerated oxidation conditions, and they have found that the development of FFA in blank treatment was faster than that of garlic-incorporated oil.

**Figure 4:**
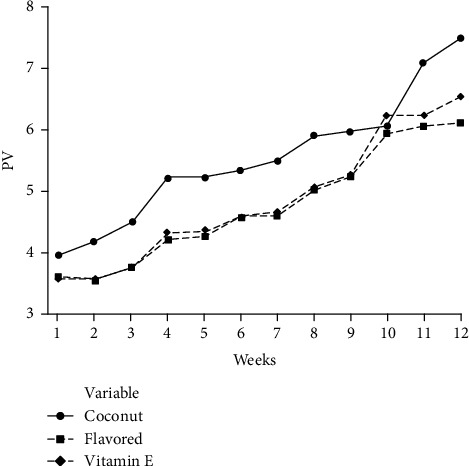
Changes of PV in coconut oil samples during storage.

The PV is the most common method of assessing the oxidative stability of vegetable oils. The amount of peroxides indicates the degree of primary oxidation, and therefore, it is linked to the rancidity. The steady increase in the peroxide value indicates the formation of hydroperoxides during the oxidation of fat. Some studies found that the incorporation of essential oil (oleoresins) into oils resulted in the least development of peroxide value [5]. This study also revealed that PV was increasing with the increment of the storage period of all oil samples (Figure 4). However, pure coconut oil samples exhibited the highest PV from the 1st week to the 12th week, and in the 12th week, it was 7.5214 ± 0.01. In the case of flavored coconut oil and vitamin E-added samples, PV were 6.1254 ± 0.01 and 6.5694 ± 0.01, respectively (at the 12-week period of storage). Further, the peroxide value was increased in all oil samples against the period of storage; however, in flavored and vitamin E-added oil samples, it was increased at a lower rate than the pure coconut oil samples (Figure 4). Since, the *p* value of pure and flavored oil samples of this study was less than 0.05 (*p* < 0.05), there was a significant difference between the PV of flavored and pure oil samples. However, since the *p* value of flavored and vitamin E-added samples was higher than 0.05 (*p* > 0.05), there were no significant differences between PV of flavored and vitamin E-added coconut oil samples.

This may be attributed to the radical scavenging efficiency of the oleoresins of spice which may lead to a decrease in the peroxide formation by neutralizing the already formed free radicals. Ramadan and Wahdan [35] have also reported that the addition of spice extracts into vegetable oil reduced the peroxide value during storage.

### 3.4. Total Phenolic Content of Flavored and Pure Coconut Oils

Determination of the total phenolic content is an important parameter to assess the benefits over certain antioxidant activity, which gives an initial insight into whether the extraction of oleoresins is worthwhile for the oil industry. The results obtained from this study were expressed as gallic acid equivalents ranging from 0.015 mg/g to 0.018 mg/g.

**Figure 5:**
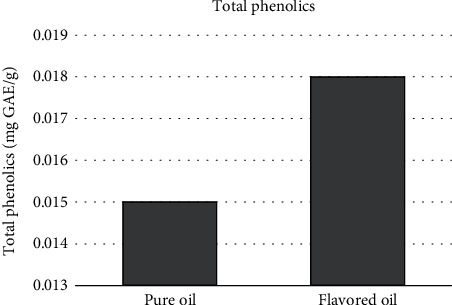
Total phenolic content of pure and flavored coconut oils.

According to the results given in Figure 5, flavored oil contained a significantly higher amount of phenolic compounds than the pure coconut oil, because *p* value for all oil samples was less than 0.05 (*p* < 0.05). The reason for this consequence is spices having a notable level of phenolic compounds. Hence, total phenolic content in flavored oil samples was higher than that of pure coconut oil. The change in the phenolic contents in flavored coconut oil was most probably due to the added oleoresins as illustrated by Figure 5.

The differences existing among the phenolic contents of unflavored and flavored coconut oils could be explained based on the interactions taking place between the oils and the flavoring agents during the mixing phase being responsible for the formation of bonds between phenolics and components of spices, herbs, and fruits [1]. McManus et al. [36] have studied the binding behavior of polyphenolics and polysaccharides of the plant cell walls and found that the molecular size of polyphenols and their conformational flexibility are important to the binding. They also noticed that small changes in the structure of either the polyphenol or the polysaccharide resulted in noticeable changes in their affinity for each other. Since phenolics are the most important naturally occurring antioxidants, the direct relation of phenolics with antioxidant capacity has been reported by Robards et al. [37], and according to Amarowicz and Pegg [38], phenolics act as dietary antioxidant, antimutagen, antiproliferative, and anticarcinogenic agents.

Kumar and Krishna [39] found that the total phenolic content of unrefined coconut has a significant difference when compared with virgin coconut oil (VCO) and RBD coconut oil. The VCO had the lowest phenolic content (1.8 mg GAE·100 g^−1^ oil) due to the removal of paring (Testa) against the RBD oil (2.1 mg GAE·100 g^−1^ oil). The difference in the total phenolic contents also affected the radical scavenging activity of the coconut oil too [39].

### 3.5. DPPH Antioxidant Activity of Flavored and Pure Coconut Oil

**Figure 6:**
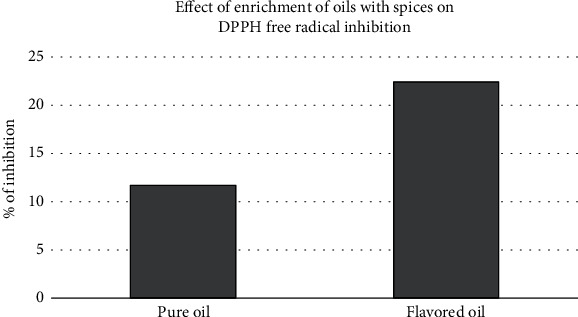
Effect of DPPH free radical inhibition of flavored and pure coconut oil.

An important parameter used to assess the antioxidant activity of flavored and pure coconut oil samples of this study was the DPPH free radical assay which has been extensively used for the characterization of the antioxidant activity of a wide range of plant material. According to Figure 6, the scavenging effects of flavored coconut oil on DPPH radicals were greater than those of pure coconut oil. As the *p* value of all oil samples was less than 0.05 (*p* < 0.05), there is a significant difference between the antioxidant activity of flavored and pure coconut oil samples. The reason for this phenomenon is the methanolic extract of spices as it contains a considerable number of phytochemicals including polyphenols that are reported to have considerably high free radical scavenging and peroxide inhibition activity [40]. Active components in spices, the concentration of the constituents, and extraction procedure are some of the important factors that affect the strength of the extract [41]. Thus, the improved oxidative stability of the treated oils can be attributed to the presence of extracted lipophilic phenolic compounds [42]. Taha et al. [43] found that only lipophilic components of the herbal materials were dissolved in the oil while leaving polar phenolic compounds undissolved because they are not fat-soluble. Hence, lipophilic components in spices are merely responsible for the antioxidant activity of the oil.

A research was conducted by Wang et al. [44] by employing 45 essential oils to determine the antioxidant activity via total phenolic content and DPPH free radical scavenging activity. They found out that the best antioxidant activity was having the cinnamon leaf oil and clove bud oil. And they mentioned that both natural and synthetic antioxidants can inhibit or postpone the process of fat oxidation. Moreover, Shahid et al. [45] also described that antioxidants can play an important role in the prevention of oxidation of fat and oil by acting as a reducing agent, free radical scavenger, chelator, and singlet oxygen scavenger.

### 3.6. GC-MS Analysis for Oils

**Figure 7:**
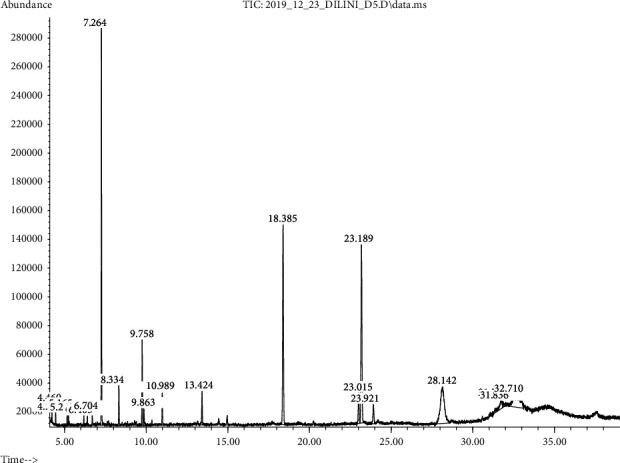
Major fatty acids and flavor compounds in flavored oil (7.264 = eugenol, 9.758 = lauric acid, 13.424 = cinnamaldehyde, 18.385 = palmitic acid, 23.189 = myristic acid, and 28.142 = piperine).

**Figure 8:**
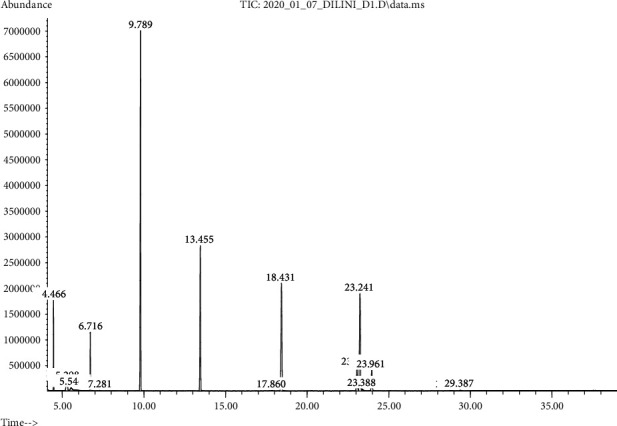
Major fatty acids in pure coconut oil (9.758 = lauric acid, 13.455 = capric acid, 18.431 = palmitic acid, and 23.241 = myristic acid).

Versatility of edible grade vegetable oils can be further expanded by augmenting them with additional important properties such as antioxidant property, shelf stability, nutritional quality, availability of bioactive compounds, and organoleptic properties along with an affordable price for the end-user. In this study too, oleoresins extracted from selected spices were incorporated into coconut oil (flavored coconut oil), and the magnitude of augmentation of bioactive compounds from oleoresins was measured in terms of the GC-MS method. According to the analysis of flavored coconut oil samples as per fatty acid methyl esters, eugenol, cinnamaldehyde, piperine, and other flavor compounds were found (Figure 7) while none of them were in pure coconut oil samples (Figure 8).

### 3.7. SPME-GC-MS Analysis for Volatile Compounds in Flavored and Pure Coconut Oil

Solid-phase microextraction (SPME) is a microsampling technique which has found wide application in flavor and fragrance research. It is a solvent-free method that is used to trap flavors and fragrances either from aqueous samples (immersion SPME) or from the vapor space above a liquid or a solid sample (headspace SPME).

**Figure 9:**
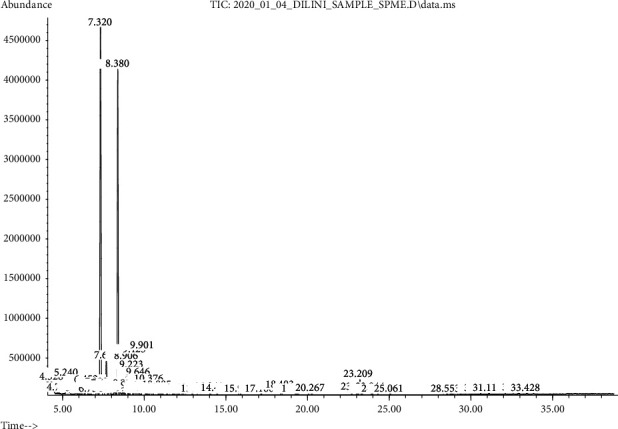
SPME results for flavored coconut oil (7.320 = phenol, 2‐methoxy‐3‐(2‐propynyl), 8.380 = caryophyllene, 7.684 = eugenol, 10.424 = cinnamaldehyde, and 28.532 = piperine).

According to the SPME analysis of flavored coconut oil samples of this study (Figure 9), the following bioactive compounds were found, namely, cinnamaldehyde, eugenol, piperine, and caryophyllene which are natural bicyclic sesquiterpenes of many essential oils, especially found in clove oil [46] and 2-methoxy-4-(2-propenyl) phenol which is a constituent of clove oil, nutmeg, cinnamon, and bay leaf [3].

## 4. Conclusions

This study was conducted to develop a spicy flavored edible coconut oil along with an extended shelf life. Coconut oil was selected for this study because it is widely used for cooking purposes. To prepare the spicy flavored coconut oil, ginger, garlic, nutmeg, black pepper, cinnamon, and clove oleoresins were incorporated into oil samples. Flavored and pure coconut oil samples of this study showed different physiochemical properties. FFA, peroxide value, saponification value, and iodine value of flavored oil were decreased relative to the same parameters of pure coconut oil. Moisture content, insoluble impurities, and the specific gravity of both flavored and pure coconut oil samples remained almost the same during the period of study. The smoke point and flashpoint of flavored coconut oil were decreased while increasing the viscosity. Since the spices containing antioxidants, the thermal stability of flavored oil samples was better than that of pure coconut oil. The increment of the FFA level and peroxide value of flavored coconut oil samples occurred at a slower rate than that of pure coconut oil samples during the study period. The shelf life of flavored oil samples was analyzed according to the FFA and peroxide value. In flavored oil, FFA and peroxide value were increased at a slower rate than the pure coconut oil samples. Shelf-life testing further revealed that oleoresins and vitamin E-incorporated samples have the same rate of increment of FFA and PV. Total phenol content and antioxidant activity of flavored oil samples were increased considerably against pure coconut oil samples. Therefore, flavored coconut oils were less susceptible to oxidation, as they are rich in antioxidants of spices. Hence, spicy flavored coconut oil is a better option for the future oil-related food industry.

## Figures and Tables

**Table 1:** Physicochemical properties of flavored and pure coconut oil samples after one week of storage.

Parameter	Pure coconut oil			Flavored coconut oil		
Peroxide value (mEq/kg)	3.989 ± 0.006			3.626 ± 0.002		
FFA value (%) as lauric acid	0.646 ± 0.001			0.604 ± 0.002		
Saponification value	249 ± 1			222 ± 1		
Iodine value	6.452 ± 0.08			5.081 ± 0.02		
Moisture (%)	0.1433 ± 0.02			0.1800 ± 0.01		
Insoluble impurities (%)	0.05 ± 0.01			0.05 ± 0.01		
Smoke point (°C)	193 ± 3			157 ± 2.5		
Flashpoint (°C)	222 ± 3			181 ± 2		
Viscosity (mPa·s)	18.633 ± 0.05			19.400 ± 0.05		
Specific gravity	0.924 ± 0.005			0.995 ± 0.005		
Color	*L*∗	*a*∗	*b*∗	*L*∗	*a*∗	*b*∗
	73 ± 2	5 ± 1	69 ± 2	67 ± 2	14 ± 1	46 ± 2

## Data Availability

The numerical data used to support the findings of this study are available from the corresponding author upon request.
